# Application of DNA Barcodes in Asian Tropical Trees – A Case Study from Xishuangbanna Nature Reserve, Southwest China

**DOI:** 10.1371/journal.pone.0129295

**Published:** 2015-06-29

**Authors:** Xiao-cui Huang, Xiu-qin Ci, John G. Conran, Jie Li

**Affiliations:** 1 Laboratory of Plant Phylogenetics and Conservation, Xishuangbanna Tropical Botanical Garden, Chinese Academy of Sciences, Kunming, Yunnan, People’s Republic of China; 2 Key Laboratory of Tropical Forest Ecology, Xishuangbanna Tropical Botanical Garden, Chinese Academy of Sciences, Menglun, Yunnan, People’s Republic of China; 3 Key Laboratory of Tropical Plant Resources and Sustainable Use, Xishuangbanna Tropical Botanical Garden, Chinese Academy of Sciences, Menglun, Yunnan, People’s Republic of China; 4 University of Chinese Academy of Sciences, Beijing, People’s Republic of China; 5 Centre for Evolutionary Biology and Biodiversity & Sprigg Geobiology Centre, School of Biological Sciences, Benham Bldg DX, The University of Adelaide, Adelaide, South Australia, 5005, Australia; Aristotle University of Thessaloniki, GREECE

## Abstract

**Background:**

Within a regional floristic context, DNA barcoding is more useful to manage plant diversity inventories on a large scale and develop valuable conservation strategies. However, there are no DNA barcode studies from tropical areas of China, which represents one of the biodiversity hotspots around the world.

**Methodology and Principal Findings:**

A DNA barcoding database of an Asian tropical trees with high diversity was established at Xishuangbanna Nature Reserve, Yunnan, southwest China using *rbc*L and *mat*K as standard barcodes, as well as *trn*H–*psb*A and ITS as supplementary barcodes. The performance of tree species identification success was assessed using 2,052 accessions from four plots belonging to two vegetation types in the region by three methods: Neighbor-Joining, Maximum-Likelihood and BLAST. We corrected morphological field identification errors (9.6%) for the three plots using *rbc*L and *mat*K based on Neighbor-Joining tree. The best barcode region for PCR and sequencing was *rbc*L (97.6%, 90.8%), followed by *trn*H–*psb*A (93.6%, 85.6%), while *mat*K and ITS obtained relative low PCR and sequencing success rates. However, ITS performed best for both species (44.6–58.1%) and genus (72.8–76.2%) identification. With *trn*H–*psb*A slightly less effective for species identification. The two standard barcode *rbc*L and *mat*K gave poor results for species identification (24.7–28.5% and 31.6–35.3%). Compared with other studies from comparable tropical forests (e.g. Cameroon, the Amazon and India), the overall performance of the four barcodes for species identification was lower for the Xishuangbanna Nature Reserve, possibly because of species/genus ratios and species composition between these tropical areas.

**Conclusions/Significance:**

Although the core barcodes *rbc*L and *mat*K were not suitable for species identification of tropical trees from Xishuangbanna Nature Reserve, they could still help with identification at the family and genus level. Considering the relative sequence recovery and the species identification performance, we recommend the use of *trn*H–*psb*A and ITS in combination as the preferred barcodes for tropical tree species identification in China.

## Introduction

Species identification is of critical importance in conserving and utilizing biodiversity, but this is often hindered by a lack of professional knowledge of classification [1]. As one of the most vulnerable floras to the increasing threats from human activities [2], tropical plant species are badly in need of rapid identification methods to aid in the development of reasonable protection strategies [3]. Unfortunately, traditional morphological taxonomy is time-consuming and dependent on pre-determined classifications and expertise [4]. Furthermore, identification is a challenge for tropical trees, even for experts, due to the frequent absence of reproductive organs that are needed to distinguish among morphologically similar species, but are often unavailable during field surveys [5]. A wide range of molecular methods have been applied to overcome this, but Hebert *et al*. [6] presented an important tool of DNA barcoding which provides a fast and effective means for species assignment without the need for detailed taxonomic expertise.

An ideal barcode should meet the need for rapid enough evolution to distinguish between species, combined with conserved regions, which will function as universal primer binding sites for PCR [7]. However, because it has proved difficult to find a single barcoding locus for plants, a combination of two or more loci is normally proposed. Recently, a consensus has emerged for using the plastid genes *rbc*L and *mat*K as standard markers to barcoding plants [8], as *rbc*L is the most effective locus for PCR amplification and sequencing [9], while *mat*K performs well for species identification in some cases [10]. In addition, there are also reports suggesting the potential of the non-coding *trn*H*–psb*A [11,12] and nuclear ITS regions [13,14] as markers and these four loci have now been utilized in numerous plant of barcoding studies [15–18].

Xishuangbanna Nature Reserve in southern Yunnan Province is located at an intermediate zone between tropical Southeast Asia and subtropical East Asia and, as such, represents the northern limit of tropical rain forest distribution in China. The region is of considerable interest to biologists for biodiversity conservation [19], containing 3,336 angiosperms from 1,140 genera in 197 families [20]. It also contains different vegetation types, of which tropical rain forest is the most common and the least threatened, whereas tropical seasonal moist forest associated with limestone habitat is considered vulnerable, mostly though habitat fragmentation due to land clearing.

In this study, we use a plot-based sampling strategy to establish a local DNA barcode database of tropical trees occurring two different vegetation types and evaluate the performance of DNA barcodes in the Xishuangbanna Nature Reserve. Specifically, we analyzed sequence recovery and species discrimination of the four barcodes *rbc*L, *mat*K, *trn*H*–psb*A and ITS singly and in combination, particularly for the following:

1. Assessment errors of morphological identification in ecological surveys based on core barcodes (*rbc*L + *mat*K);
2. Comparison of sequence recovery of the four selected markers between our study and other comparable studies;
3. Evaluating species resolution for different methods with various barcodes combinations;
4. Comparison of the ability of species identification between this study and others studies based on plant DNA barcoding with geographically bounded sampling;
5. Evaluate the ability of DNA barcoding in this region.

## Materials and Methods

### Ethics statement

All the fieldwork was conducted at Xishuangbanna Nature Reserve under permit issued by the Forestry Department of Yunnan Province and Xishuangbanna National Nature Reserve Administration and collecting procedures were done with proper precautions for minimizing impacts to protected or endangered trees in these areas. We stated field studies did not involve any locations for which no specific permission was required.

### Study site and sampling

Fieldwork in this study was conducted from 2008 to 2012. Four plots established by the Xishuangbanna Tropical Rainforest Ecology Station (XSTRES) were selected on basis of vegetation type and different level of ecological surveys (named the 20 ha Xishuangbanna tropical seasonal rainforest dynamics plot (BB), JJYL, GGYL and LSL) (Table 1). There are two vegetation types—tropical rain forest (BB, JJYL and GGYL) and tropical seasonal moist forest (LSL) in our study [19]. The 20 ha permanent dynamic plot was established in 2007 following the protocol for large forest dynamics plot of Center for Tropical Forest Science [21]and tree species were identified by ecologists and taxonomists. Other three plots were identified in the field by ecologists. We collected mature leaves from 1–6 individuals for each tree species ≥1cm of diameter at breast height and dried with silica gel. Cambium (bark) tissues were also used as an alternative for canopy trees if they were too tall for leaf collection. Voucher specimens were collected and deposited at the herbarium of Xishuangbanna Tropical Botanical Garden (XTBG), Chinese Academy of Science (CAS).

**Table 1:** Sampling information of four plots in the Xishuangbanna Nature Reserve.

Plots	Square metre	Total individuals	Total species	Selected individuals for experiment	Selected species for experiment
BB	400×500	93,410	468	1019	357
JJYL	100×100	5149	351	725	304
GGYL	100×50	2083	137	220	116
LSL	50×50	1455	47	88	44

### DNA isolation, amplification and sequencing

Total genomic DNA was isolated from approximately 30 mg of dried leaf or cambial material using the Plant Genomic DNA Kit (Tiangen Biotech Co., China), either according to the manufacturer’s protocols, or modified as needed. For example, extraction with chloroform isoamyl alcohol (24:1) was repeated twice when the material was rich in secondary metabolites.

We amplified chloroplast regions *rbc*L, *mat*K, *trn*H*–psb*A and the nuclear region ITS using multiple primers with broad taxonomic versatility. As standard barcodes, *rbc*L and *mat*K are used widely and recommended due to high amplification levels in plants [22, 23], thus, these two barcodes are especially helpful for mass screening data. Similarly, ITS and *trn*H*–psb*A showed considerable utility for species identification [24]. For *mat*K, four primer sets were tested, due to its generally poor performance of amplification and sequencing [25]. All PCR reactions had a total volume of 25 μL and DMSO and BSA were added to enhance the PCR performance for *mat*K and ITS. In order to test the effects of different PCR procedures on sequence recovery, we conducted two sets of cycling conditions (general and Ramp procedures) applied to samples from the three small plots (JJYL, GGYL and LSL). For primer combinations, PCR thermal conditions and references see S1 Table. All the PCR products were sequenced at the Beijing Genomics Institute (BGI).

### Sequence editing and alignment

We assembled consensus sequences using Sequencher 4.14 (GeneCodes Corp., Ann Arbor, MI, USA) and aligned them with different programs (*i*.*e*. ClustalW [26], MUSCLE 3.8.31 [27] and SATé [28]). For two core markers (*rbc*L, *mat*K) and the nuclear marker ITS, a global multiple sequence alignment was used. The *rbc*L sequences were unambiguous due to the absence of insertion or deletion. Alignment of *mat*K was more difficult due to the insertion of triplet codons, so we checked the alignment results visually. Both *rbc*L and *mat*K were aligned several times by ClustalW and MUSCLE. Because the ITS sequences were more difficult to align, we used the Simultaneous Alignment and Tree Estimation (SATé) for global multiple alignment (http://phylo.bio.ku.edu/software/sate/sate.html). Similarly, the *trn*H*–psb*A sequences were highly variable and could not be handled with a global multiple sequence alignment. As a result, we conducted a family-based alignment using ClustalW and then created a supermatrix by concatenating them with the aligned sequences of the other markers [29].

### Detecting errors in tropical tree identification

It is difficult to identify woody plants in the tropics because most of the trees encountered in the field are not reproductive at the time of sampling and must be identified using vegetative characters, but most species descriptions and keys rely on flower and fruit characters [30], often resulting in misidentification of sterile material. Here, we adopted a two-step procedure of reciprocal illumination, combining morphology and DNA sequence data to uncover and correct mistakes in species identification in three plots (JJYL, GGYL and LSL), in which the morphology-based identifications were undertaken in the field. Firstly, we detected potential errors through examination of the Neighbor-Joining trees using the core barcodes. Secondly, we reviewed the morphology of the species involved, comparing specimens with relevant herbarium vouchers from other studies, to confirm whether mistakes based on morphological identification had been made. DNA extraction and subsequent trials were also repeated when herbarium vouchers were absent, until all the samples were considered to be error free.

### Data analysis

There are numerous methods used for the analysis of barcode data and species resolution, of which phylogenetic analysis [17,31–33] and similarity approaches such as BLASTn [8,34] are the most commonly used for DNA barcode data analysis. The similarity-based BLASTn is an algorithm for comparing query sequences with reference database calculating pairwise alignments in the process. All sequences in our study served as both database and query and were queried individually to the database. Additionally, we also conducted stand-alone BLAST comparisons, only using the sequence database of the BB plot, where the most species occurred. All barcodes were tested singly and in combination. We considered an assignment to be correct when query sequences showed ≥95% identical sites to sequences of the same species in the database and all the sequences of the species showed higher identical sites compared with sequences for other taxa.

We also present results based on Neighbor-Joining and Maximum-Likelihood trees, because some studies have shown that different algorithms for reconstructing trees did not alter the performance of DNA barcodes significantly [35,36]. We tested to see if the individual species were retrieved as monophyletic groups for each barcode locus and their different combinations. The NJ tree reconstruction was constructed using Geneious 6.1.6, while the ML analysis was conducted using RAxML [37] via the CIPRES supercomputer cluster (www.phylo.org). Bootstrap analyses were based on 1000 replicates for NJ trees and 100 for ML trees. For a given barcode locus or combination of loci, we used a cutoff of 50% to define support for “successful” resolution of monophyletic species [38].

### Testing the barcoding accuracy at the regional scale

To determine if species identification success was lower between different plots than within them, we established a barcoding database from the BB plot. For the other three plots (JJYL, GGYL and LSL), only those individuals that belonged to taxa present in the BB plot were used for the regional scale analysis. To this end, we selected all the samples from the three plots that belonged to a species or genus represented by at least one individual in the database from BB plot, using the BLAST method to assign a species or genus to the specimens in the three smaller plots with the BB specimens as reference database.

## Results

### PCR and sequencing success rates

In total, we obtained 5583 sequences from 2052 samples, representing 655 species, 259 genera and 76 families. These included 1654 sequences for *rbc*L, 1430 sequences for *trn*H*–psb*A, 1422 sequences for *mat*K and 1077 sequences for ITS (Table 2). We recovered one sequence for at least one of the four markers in 1858 (90.5%) samples; however, 194 samples failed for all four regions. *rbc*L showed the highest PCR and sequencing success rates of 97.6% and 90.8%, respectively. The next best PCR and sequencing rates were exhibited by *trn*H*–psb*A (93.6%, 85.6%) and *mat*K (89.5%, 79.5%), followed by ITS (86.2%, 71.0%). The two chloroplast genes (*mat*K, *trn*H*–psb*A) gave poorer PCR results for the BB plot than the other three plots (JJYL, GGYL, LSL). In *mat*K, the PCR success rate for the BB plot was only 80.7%, while for the other three plots were all over 90%. Similarly, the PCR success rate of *trn*H*–psb*A in the BB plot was 89.3%, but the other three plots gave rates over 95% (Table 2).

**Table 2:** PCR amplification and sequencing success of the four plots in Xishuangbanna.

	Total individuals for experiment		PCR amplification success			Sequencing success			
DNA barcodes		*rbc*L	*mat*K	*trn*H*–psb*A	ITS	*rbc*L	*mat*K	*trn*H*–psb*A	ITS
**BB**	1019	97.00%	80.70%	89.30%	87.50%	96.30%	76.80%	87.50%	77.10%
**JJYL**	725	99.60%	99.20%	97.70%	81.20%	83.60%	83.00%	85.10%	60.60%
**GGYL**	220	95.00%	96.80%	98.20%	94.10%	89.10%	79.50%	80.50%	76.40%
**LSL**	88	94.30%	94.30%	98.90%	93.20%	90.90%	81.80%	80.70%	73.90%
**Total**	2052	97.60%	89.50%	93.60%	86.20%	90.80%	79.50%	85.60%	71.00%

### Mistakes in taxonomic identification

Based on Neighbor-Joining analyses, 99 individuals (9.6%) were misidentified morphologically from the JJYL, GGYL and LSL plots (S1 and S2 Figs). Excluding the seven unknown individuals, we found that out of the remainder, 70 samples were misidentifications at the family level, while 17 and five samples were at the genus and species level, respectively. Comparing morphology-based identifications and corrected identification results derived from DNA sequences, we only observed seven cases in which all individuals of a certain species were mistaken for an another species. These were all cases of morphological convergence in vegetative characters e.g.: *Chrysophyllum lanceolatum* (Blume) A. DC. versus *Ardisia scalarinervis* E. Walker and *Epiprinus siletianus* (Baill.) Croizat versus *Ixora amplexicaulis* C.Y. Wu & W.C. Ko. However, most of the errors were found to be mistakes in individual sample identifications, resulting in some individuals of one species nesting with those of another.

### Species resolution: single-region analysis

We conducted species identification analysis based on the corrected results derived from the reciprocal illumination procedure. The performances of the four markers using the three barcoding identification methods within our four plots in Xishuangbanna provided relatively similar results, although in this study, *trn*H*–psb*A could not be evaluated by analysis of reconstructing trees because we could not obtain good results from global multiple sequence alignments due to variations among such diverse taxonomic groups, mainly due to high numbers of insertions and/or deletions [31].

**Fig 1:**
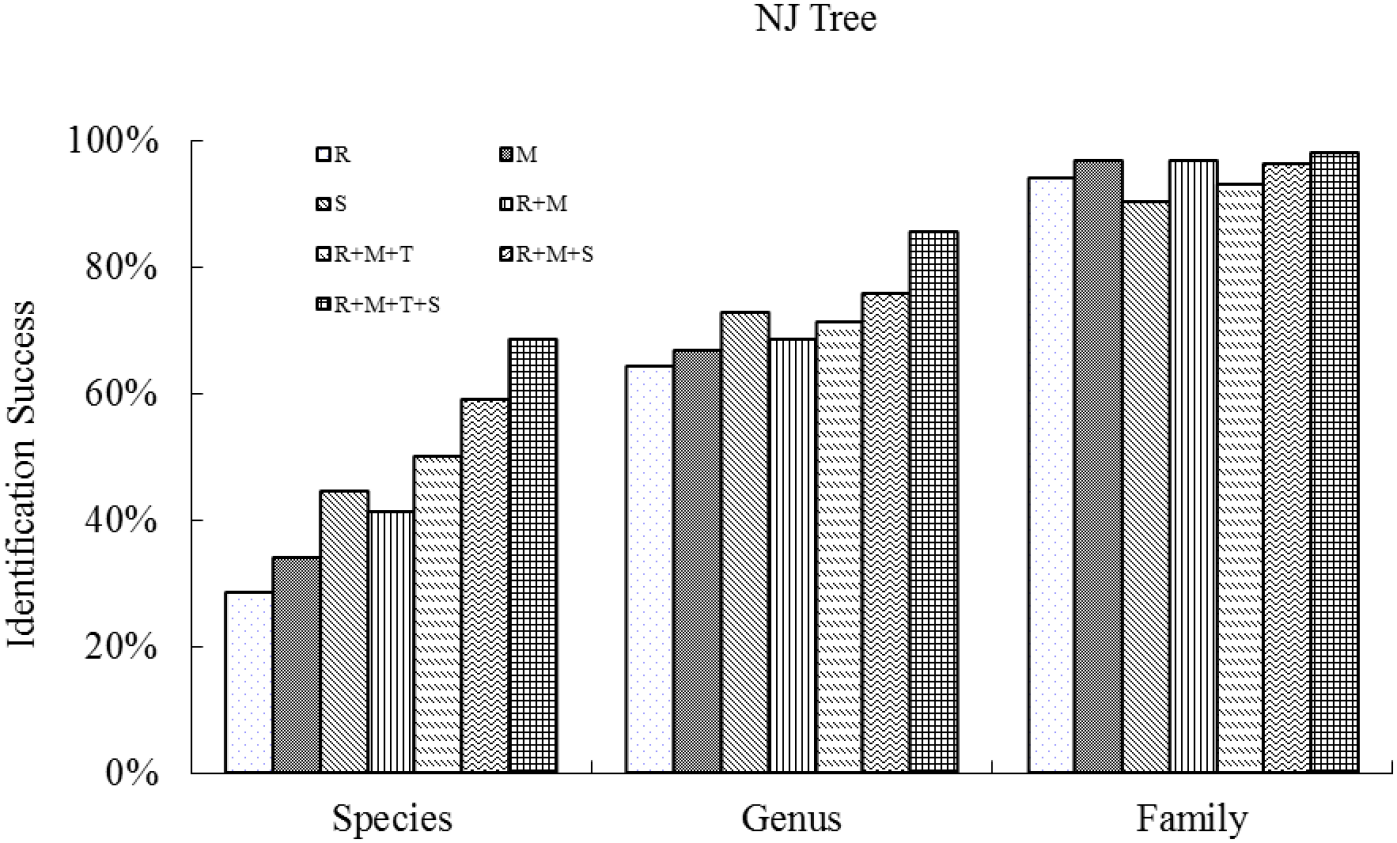
Species resolution success at the family, genus and species levels for single regions and combinations, based on Neighbor-Joining Tree analysis of all the species (samples ≥ 2), collected from the four plots (BB, JJYL, GGYL and LSL) of Xishuangbanna Nature Reserve in southwest China. (R, M, T, S represent *rbc*L, *mat*K, *trn*H–*psb*A and ITS respectively.)

**Fig 2:**
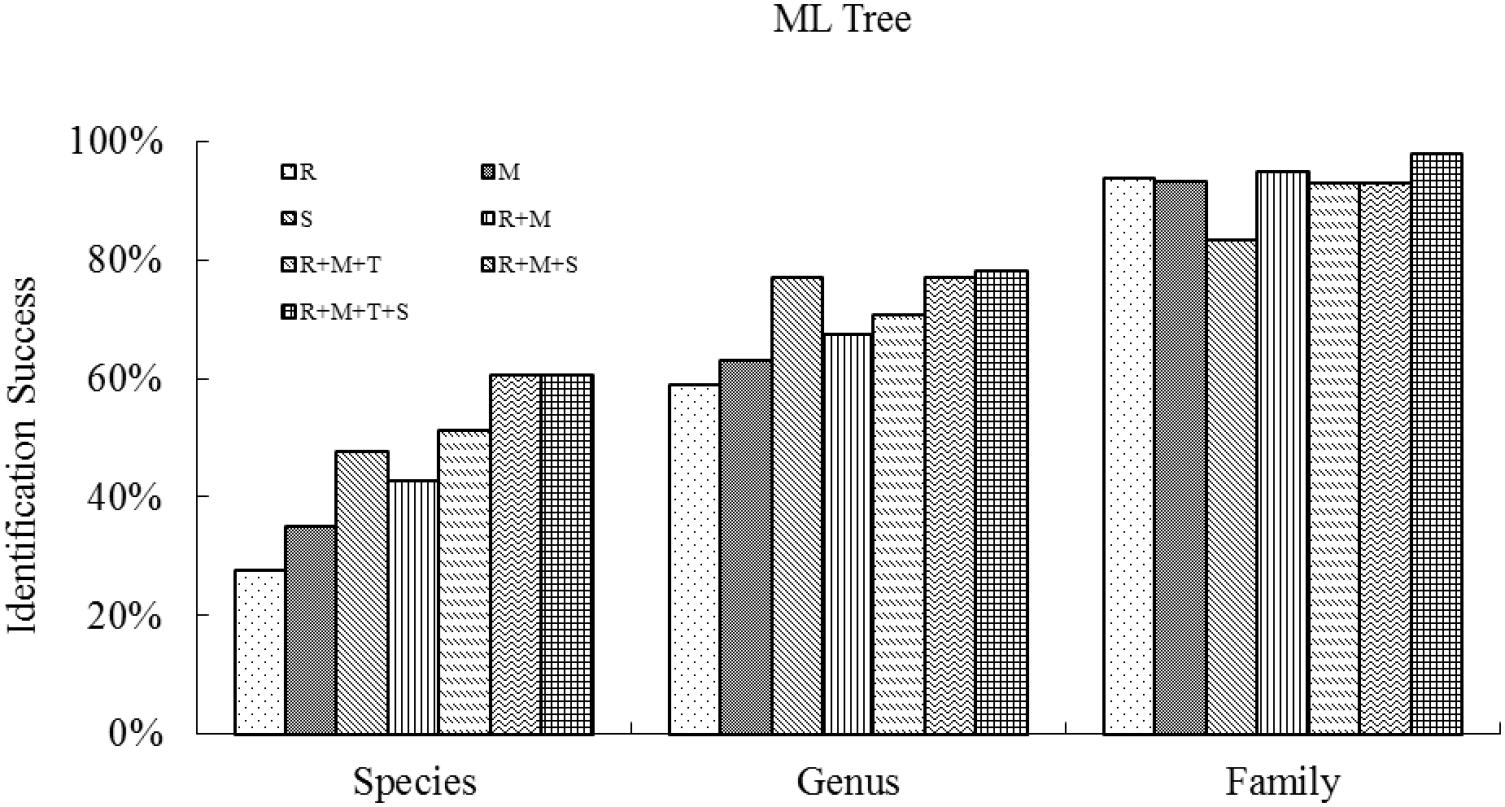
Species resolution success at the family, genus and species levels for single regions and combinations, based on Maximum Likelihood Tree analysis of all the species (samples ≥ 2), collected from the four plots (BB, JJYL, GGYL and LSL) in the Xishuangbanna Nature Reserve in southwest China. (R, M, T, S represent *rbc*L, *mat*K, *trn*H–*psb*A and ITS respectively.)

The highest success of species discrimination based on two tree building methods (NJ and ML) with single barcode were obtained with ITS (44.6% and 47.8%), followed by *mat*K (34.1% and 35.3%) and then *rbc*L (28.5% and 27.8%). At the genus level, the results also performed best with ITS (72.8% and 77.2%), followed by *mat*K (66.7% and 63.1%) and *rbc*L (64.3% and 59.1%) (see Figs 1 and 2).

**Fig 3:**
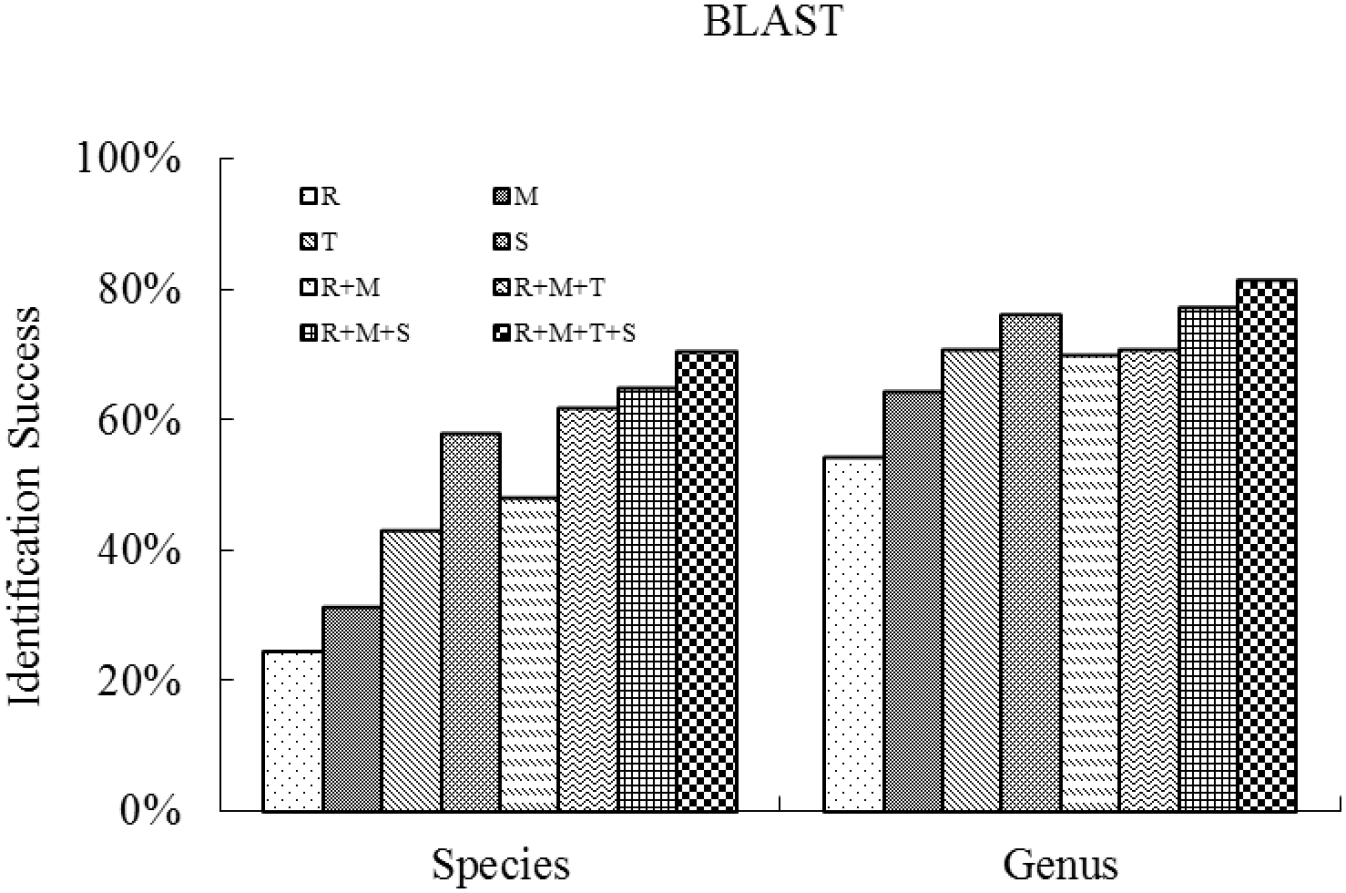
Percent species resolution at the genus and species levels for single regions and combinations, based on BLAST analysis of all the samples, collected from the four plots (BB, JJYL, GGYL and LSL) of Xishuangbanna Nature Reserve in southwest China. (R, M, T, S represent *rbc*L, *mat*K, *trn*H–*psb*A and ITS respectively.)

Of the three methods, BLASTn tended to show slightly higher discrimination success rates for the four genes. For all the 1858 samples for which we obtained at least one sequence, percent species-level resolution ranged from 58.1% (ITS) to 24.7% (*rbc*L) with *trn*H*–psb*A and *mat*K having intermediate values of 43.4% and 31.6%, respectively. Similar patterns were observed for genus-level resolution; ITS again providing the highest genus discrimination success rate (76.2%) and *rbc*L the lowest (54.5%), while *trn*H*–psb*A (70.9%) and *mat*K (64.4%) were intermediate. Thus, percentage species and genus resolution were higher with the two supplementary barcodes compared with the two core barcodes among the three analysis methods in our study. However, the four genes were all prone to higher species discrimination when we just used the samples of BB plot to manage a stand-alone BLAST. The species-level identification success rates for *mat*K, *rbc*L, *trn*H*–psb*A and ITS were 60.0%, 61.3%, 79.7% and 84.7% respectively, with genus-level success rates for *rbc*L, *mat*K, *trn*H*–psb*A and ITS of 79.8%, 84.4%, 94.6% and 95.3% (Fig 3).

### Species resolution: multi-region analysis

We found little difference between the two methods of phylogenetic tree reconstruction (NJ and ML) for the different barcode combinations (Figs 1 and 2) as follows: *rbc*L + *mat*K (41.3% and 42.9%), *rbc*L + *mat*K + *trn*H*–psb*A (50.0% and 51.3%), *rbc*L + *mat*K + ITS (58.9% and 60.8%) and *rbc*L + *mat*K + *trn*H*–psb*A + ITS (68.6% and 60.7%).

The *rbc*L + *mat*K barcodes identified 48.4% of the species we sampled in the four plots using BLASTn (Fig 3). The addition of a non-coding region to this combination increased resolution by 13.5% (to 61.9% for *rbc*L + *mat*K + *trn*H*–psb*A) and a nuclear region by 16.7% (to 65.1% for *rbc*L + *mat*K + ITS) and the addition of all four gene regions resulted in 70.6% species resolution. The genus-level resolutions across all four barcode combinations ranged from 70.2% (*rbc*L + *mat*K) to 81.6% (*rbc*L + *mat*K + *trn*H*–psb*A + ITS).

### Barcoding accuracy at the regional scale

**Fig 4:**
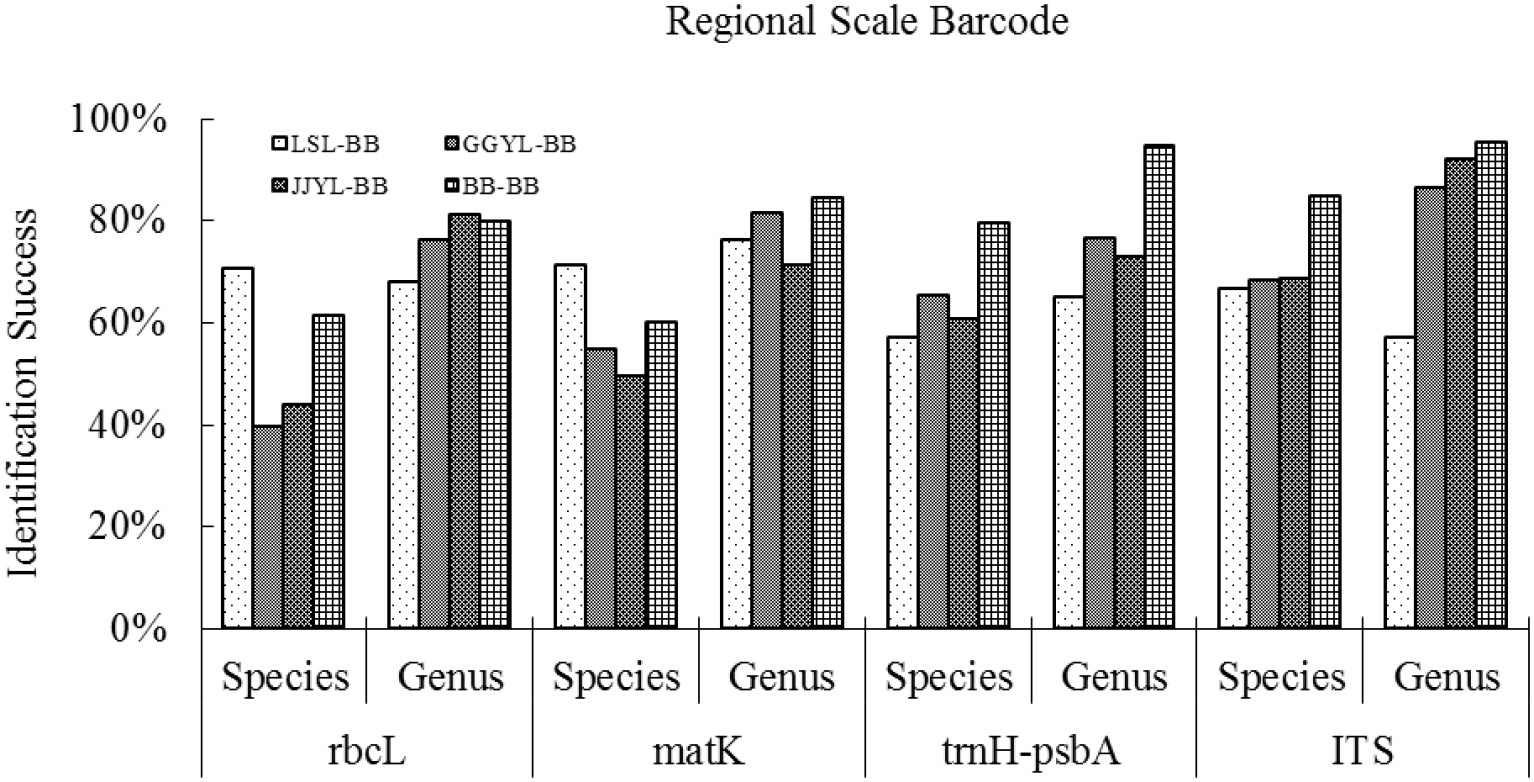
Barcoding success of Xishuangbanna tropical trees at a regional scale for species identification and genus identification. (LSL-BB, GGYL-BB, JJYL-BB, BB-BB mean that the samples of BB serve as a database, while the samples of LSL, GGYL, JJYL and BB as queries respectively.)

At the regional scale, we tested the effectiveness of BLAST for species and genus identification. Here, all samples from the dataset in the three small plots for species or genera identification should be present in our database of barcode sequences from the BB plot (Table 3). For the JJYL plot, identification was most successful with ITS at the species level (68.6%), followed by *trn*H*–psb*A (60.7%), *mat*K (49.5%), then *rbc*L (44.0%). The genus-level identification success of the JJYL sequences reached 92.2% using ITS, 81.1% with *rbc*L, 73.0% with *trn*H*–psb*A and 71.2% with *mat*K. The poorest performing barcode locus in the GGYL sequences was *rbc*L for both species- and genus-level discrimination, while the best was ITS. In contrast, in the LSL plot the performance of all four barcodes was different from the JJYL and GGYL plots, with the two core barcodes (*rbc*L, *mat*K) showing higher discrimination success rates than the *trn*H*–psb*A and ITS sequences (Fig 4).

**Table 3:** Shared numbers of species and genera among the four plots (BB, JJYL, GGYL and LSL).

Shared species (genera) for *rbc*L /*mat*K /*trn*H*–psb*A */* ITS	LSL	GGYL	JJYL
**LSL**			
**GGYL**	8 (9) /6 (6) /7 (8) /1 (2)		
**JJYL**	17 (19) /13 (17) /17 (18) /6 (11)	77 (53) /65 (49) /76 (53) /44 (38)	
**BB**	17 (25) /14 (21) /14 (20) /9 (14)	58 (55) /42 (43) /55 (51) /38 (37)	125 (122) /109 (104) /112 (111) /86 (90)

## Discussion

Our study is the first attempt to barcode a local tropical tree flora from the Xishuangbanna Nature Reserve in Southwest China. The creation of our study provides a local platform for a broad range of applications that are reliant on large-scale species identification.

### Sequence recovery

Our results showed higher sequence recovery for *mat*K, compared with only 42% in one case [39] and around 70% in others [40,41,42], Fazekas *et al*. [43] reported a higher level of success (88%) for *mat*K using 10 primer pairs, while sequencing success of 90% was obtained by the CBOL Plant Working Group using two primer pairs [24], which is similar with our results. This difference in relative performance may be explained either by the choice of primer combinations and/or the numbers of primer pairs used. Compared with other DNA barcode studies of tropical trees (e.g. 42% amplification success and 27% sequencing success for *mat*K using three primer pairs in India [44]), our results for *mat*K were much higher using only four primer pairs. This could have resulted from more sophisticated amplification, for example, Ford *et al*. (2009) obtained 85% success rate with *mat*K using a combination of standard and nested multiplexed-tandem PCR (MT-PCR) [39]. In this study, we also conducted a modification of the cycling procedure named Ramp-PCR for each of the three chloroplast regions in the three smaller plots, resulting in higher PCR amplification and sequencing success for the three chloroplast genes and especially *mat*K for these areas relative to the BB plot. Basic PCR programs were used during experiments of the BB plot, which were finished in the early 2009 at the beginning of DNA barcoding project in Xishuangbanna. Later, for the other plots, we attempted to use ramp-PCR. We consider that these observed differences in site-level barcoding success were due mainly to the combination of standard and Ramp PCR process used in the three small plots. As a result, we suggest that the additional cost of testing a large number of primer combinations of chloroplast genes might be less cost-effective than implementing more suitable, but non-standard PCR methods when conducting a DNA barcode study involving large numbers of phylogenetically diverse and genetically variable plant species.

The PCR amplification and sequencing success rates of the non-coding gene *trn*H*–psb*A region ranked only second to *rbc*L. This was in accordance with several other studies implying that the resolution of *trn*H*–psb*A was high enough to be considered as a barcode [7,8,36,45]. Although some studies have criticized the use of this marker because it is considered likely to develop errors during sequencing [46], we obtained high quality contigs with a success rate of 85.6%, which was nearly as good as *rbc*L, which is generally considered to be one of the most efficient barcode loci for plants [24,27]. Due to the high numbers of substitutions seen in *trn*H*–psb*A, it has the potential to be a suitable marker for discrimination between closely related species [47]. This barcode gene was recommended as one of the best performing locus for barcoding tropical tree species [35] and in terms of PCR amplification success, sequencing and species resolution, we would support this.

The only nuclear barcode gene in our study provided the highest species resolution ability among of the four tested regions using both tree-based and stand-alone BLAST methods in each of the four plots. ITS has been shown to discriminate species in many groups [14,16,48,49]; however, although we obtained an acceptable PCR amplification success rate of 86.2% for ITS, there was still a relatively low overall sequencing success rate of 71.0%. Some other studies have reported difficulties of sequencing ITS because of issues relating to secondary structure formations in this region [50,51]. Nevertheless, despite the lower sequence recovery by ITS compared to the other three markers at Xishuangbanna, we still obtained higher sequence recovery than some other DNA barcoding studies of tropical forests (41.0% for Amazonia [5] and 62.0% for India [44]). Therefore, we would support the use of ITS as a barcode marker for tropical tree species in Xishuangbanna.

### Reducing mistakes in taxonomic identification

A tree-based approach using DNA barcoding in combination with morphology is very useful to revise mistakes of morphological identification [12]. We recommended the core barcodes-*rbc*L and *mat*K to do this work for two reasons. First, the high rates of sequence recovery make it possible to find identification errors from as many samples as possible using the core barcodes. Second, data analysis is easy for the core barcodes because they are coding regions, for example, multiple sequence alignments. In contrast, it is difficult to assess error rates using ITS or *trn*H*–psb*A due in part to the high rate variation of sequences among such a large numbers of taxa, in contrast to work on the tropical tree *Inga* Mill. (Fabaceae), where the focus of that study was genus-specific [30]. From this perspective, it was less economical to detect error identifications or assign unknown samples to a certain taxon using the two complementary barcodes which were difficult to amplify, sequence and align among such a large numbers and variable taxa of tree species in tropics.

### Discrimination

In this study, tree-based methods performed less well for identification than similarity-based methods using BLAST. This finding is also in agreement with the results of other studies comparing relative performance of DNA barcoding methods [13,52,53], probably because tree-based methods combine all sites and/or attempt to consider relationships among the species sampled, whereas the BLAST-based approaches use local comparisons among sequences, making them more sensitive to small differences among taxa [52].

Using the samples from all four plots in this study, we found that there was relatively low species identification success for the two core barcode regions, either alone (28.5% for *rbc*L and 35.3% for *mat*K at the best), or in combination (48.4%) based on both tree-based and BLAST methods. These results are similar to previous findings for Indian tropical forests (*rbc*L 39.1%) [24] and suggest that the recommended standard barcode markers *rbc*L and *mat*K may not always be suitable for tropical tree species discrimination at the species level. Before we assessed the performance of species identification, we corrected some apparent morphological identification errors only based on *rbc*L and *mat*K which were amplified and sequenced more easily; detecting an overall 9.9% error rate in taxonomic identification using *rbc*L and *mat*K: 77.1% (74/96) at the family level, 17.7% at the generic and 5.2% at the species level. This indicates that these two core barcode genes were still useful for detecting and correcting morphology-based identification mistakes in tropical Asian tree species at the family level, which makes them useful for tropical ecological research (e.g. for investigating phylogenetic communities).

The best results for species-level identification were gained by ITS (58.1%), followed by *trn*H*–psb*A (43.4%) in BLAST. The addition of each of the two barcodes to the combination (*rbc*L + *mat*K) increased identification success from 48.4% to 61.9% by *trn*H*–psb*A and 65.1% by ITS. The three loci combinations of *rbc*L + *mat*K + *trn*H*–psb*A and *rbc*L + *mat*K + ITS provided slightly higher species resolution as that of the single loci of *trn*H*–psb*A and ITS or their combination, but the two barcode combination was more preferred here than three loci combinations in consideration of cost effectiveness. A number of studies relying upon *trn*H*–psb*A alone [16] or in combination with other regions [54–56] have verified the utility and efficacy of this region for plant DNA barcoding. The high species resolution ability of ITS was tested and compared with other candidate regions in a barcoding context [18,57,58]. Though a suitable processing method of *trn*H*–psb*A and the low sequencing success of ITS needs to dealt with, we suggest both *trn*H*–psb*A and ITS as potential genes for tropical trees in the present study. This is in line with several previous studies [5,43,52,59].

One of the important observations was the relatively low species identification by all the four loci compared with earlier similar studies in tropical areas [5,24,29,60], prompting an investigation of this poor performance in our study. We considered the taxon proportion of different areas as one of the reasons for the discrimination performance. Species resolution in Panamanian forests reached up to 98% using *trn*H*–psb*A [29], while Xishuangbanna showed the poorest species discrimination (47.8%), with intermediate values for studies in Cameroon (84.3%) [60], Amazonia (64.0%) [5] and India (60.0%) [24]. The ratios of individual/genus were Panama: 5.7 (1035/296), Cameroon: 4.9 (772/159), Amazonia: 7.5 (1073/143), India: 3.66 (300/82) and Xishuangbanna: 7.9 (2052/259), while the matching ratios of species/genus for these regions was 1.63 (296/181), 1.71 (272/159), 1.77 (254/143), 1.82 (149/82) and 2.5 (655/259), respectively (see Table 4).

**Table 4:** Comparison of relationships between the ratio (individuals/genera or species/genera) and species identification success in different tropical areas.

						Species identification success				
Area	Genera	Species	Individuals	I/G	S/G	*rbc*L	*mat*K	*psb*A*-trnH*	ITS	Literature Resource
Panama	181	296	1035	5.7	1.63	99.0%	75.0%	95.0%		Kress *et al*., 2009 [29]
Africa	159	272	772	4.9	1.71	71.2%	75.5%	84.3%		Parmentier *et al*., 2013 [60]
Amazon	143	254	1073	7.5	1.77	57.0%	61.0%	64.0%	66.0%	Gonzalez *et al*., 2009 [5]
India	82	149	300	3.7	1.82	39.1%		60.0%	74.3%	Tripathi *et al*., 2013 [24]
China	259	655	2052	7.9	2.50	28.5%	35.3%	47.8%	58.1%	This paper

I/G = individuals/genera, S/G = species/genera

A decreasing tendency in successful species identification was apparent when clade richness (species/genus) increased, yet the success rate was not affected by the number of samples per species. This result was in accordance with the research of Cameroon by Parmentier *et al*. [60]. This may also reflect the different generation times or mutation rates for the woody species in these areas, possibly contributing to the differences in species discrimination success rates [61,62].

This study showed barcode identification success with two data sets: one comprising samples with all sequences from taxa common to the four plots (BB, JJYL, GGYL and LSL) and the other, for all samples from the BB plot. Our species identification success rates for *rbc*L (61.3%) and *mat*K (60.0%) for the BB plot alone were much closer to results from Amazonia (57.0% for *rbc*L and 61.0% for *mat*K) using BLAST, while the results (79.7%,) of the supplementary barcode gene *trn*H*–psb*A performed at a level comparable to the Cameroonian rainforests of Africa (84.3%). The BB plot also displayed higher species resolution than the combined samples of the four plots in our study. In addition, we conducted a regional scale barcoding that involved the three small plots, JJYL, GGYL and LSL serving as query plots. The whole consequences using BLAST with the three small plots were lower than that with the BB plot itself and the results for JJYL and GGYL were much closer to each other than to LSL. This may have been a result of the similar vegetation tropical rainforest shared between JJYL and GGYL, whereas LSL consists of tropical seasonal moist forest.

**Fig 5:**
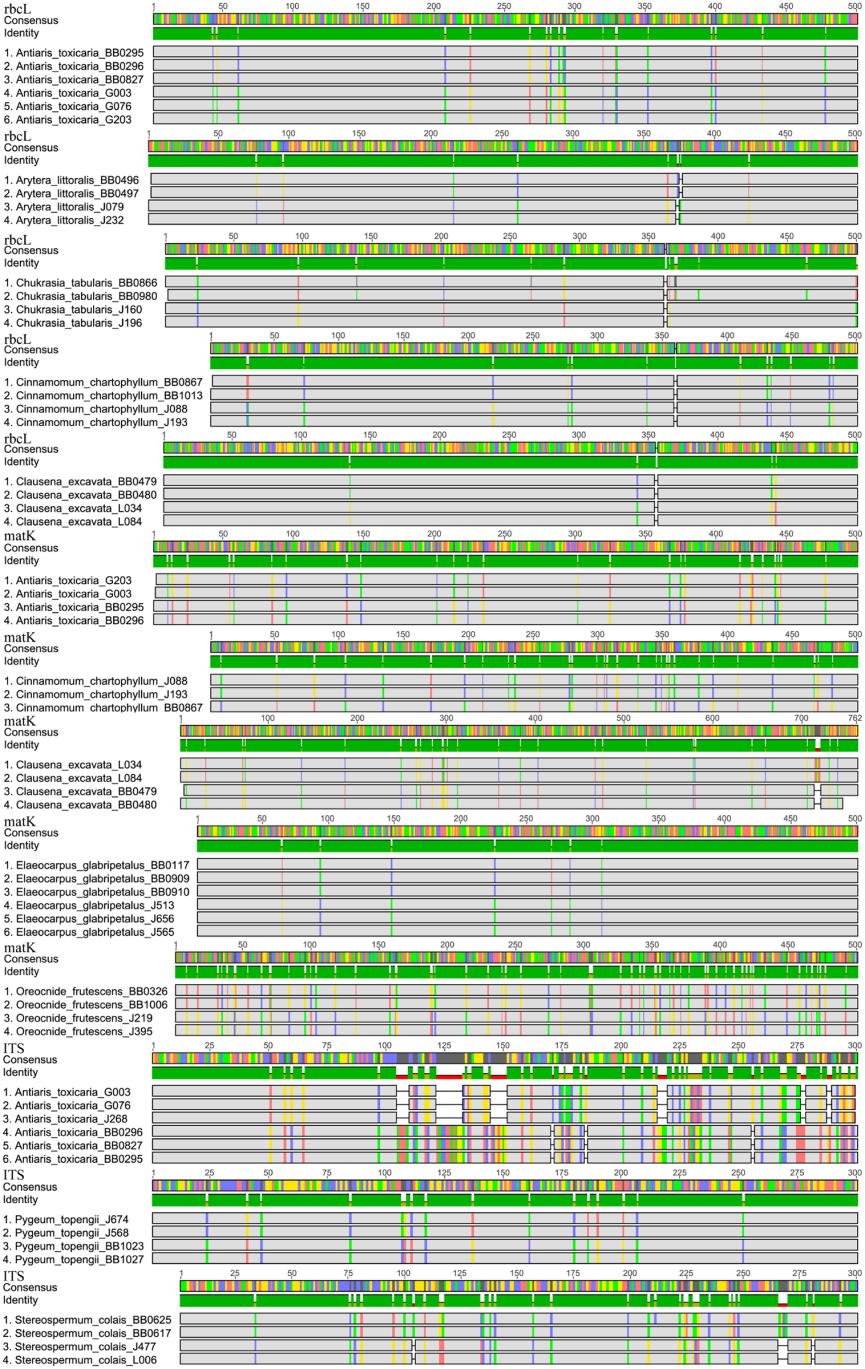
Intraspecific single base difference among the four plots (BB = BB, JJYL = J, GGYL = G, LSL = L) in Xishuangbanna Nature Reserve. (Gray = No Base Difference, Red = A, Green = T, Yellow = G, Blue = C)

These outcomes indicate an increase of genetic diversity from the local scale (single point of sampling) to the regional scale (multi-point of sampling) [63] and we did observe more intraspecific base substitutions when considering all samples in the four sites (Fig 5). Thus, the multi-point sampling strategy used here resulted in more variable intraspecific sequences, especially between different vegetation types, lowering species identification success.

## Conclusions

This study is an initial assessment of barcoding tropical tree species within the Xishuangbanna Nature Reserve, southwest China. It demonstrated that in this area, there are ecological applications for identifying invasive species [33,64], construction of phylogenetic trees for community ecology [29,41] and evaluation of the effect of species identification errors on ecological theories [30].

Large-scale biodiversity inventories are based on accurate species identification. Unfortunately, errors are common for tropical trees, usually due to the lack of reproductive characters. DNA barcoding could quickly and effectively help to correct morphological identification errors [30]. Compared with the core DNA barcodes r*bc*L and *mat*K, the species-level identification results for *trn*H*–psb*A and ITS in this study were more successful and we recommend using these two barcodes in combination as the preferred barcodes for tropical tree species in southwest China.

## Supporting Information

S1 Fig Neighbor-Joining (NJ) tree generated using *rbc*L sequences from the JJYL, GGYL and LSL plots. Error identifications at the family, genus, species levels and unknown species were highlighted in blue, green, red and purple respectively.
(EPS)

S2 Fig Neighbor-Joining (NJ) tree generated using *mat*K sequences from the JJYL, GGYL and LSL plots. Error identifications at the family, genus, species levels and unknown species were highlighted in blue, green, red and purple respectively.
(EPS)

S1 Table The systems and reaction process of PCR amplification. (DOC)
