# Supplementary material for: Application of DNA Barcodes in Asian Tropical Trees – A Case Study from Xishuangbanna Nature Reserve, Southwest China
Source: PLoS One. 2015 Jun 29;10(6):e0129295. doi: 10.1371/journal.pone.0129295 (PMC4509572; doi:10.1371/journal.pone.0129295)
Supplement: S1 Table — (DOC) [file pone.0129295.s003.doc]

**Table S1. The systems and reaction processes of the PCR amplification protocols used for barcoding**

| *rbcL* | PCR Reaction System | 20 μL: 2.0 μL 10×PCR Buffer, 2.0 μL MgCl_2_ (25mM), forward and reverse primers 1 μL (10 μM) each, 2 μL dNTPs (2.5mM), 2 μL DNA and 0.2 μL rTaq |
| --- | --- | --- |
|  | PCR Reaction Process | General Procedure: 95℃, 4 min; 5× (94℃, 30s; 53℃, 1 min; 72℃, 1 min); 30× (94℃, 30s; 52℃, 1 min; 72℃, 1 min); 72 ℃, 10 min; 16℃, end |
|  |  | Ramp Procedure: 94℃, 1 min; 5× (94℃, 30s; 65℃, 1s; 50℃, 5s; 68℃, 2 min); 30× (94℃, 30s; 64℃,1s; 50℃, 5s; 68℃, 2 min); 68℃, 1 min; 16℃, end |
|  | Primers | rbcL1F: 5’-ATGTCACCACAAACAGAGACTAAAGC-3’(Fay et al. 1997)  rbcL724R: 5’-TCGCATGTACCTGCAGTAGC-3’ (Olmstead et al. 1992)  rbcLa-F: 5’-ATGTCACCACAAACAGAGACTAAAGC-3’ (Levin 2003)  rbcLa-R: 5’-GTAAAATCAAGTCCACCRCG-3’ (Kress et al. 2009) |
| *matK* | PCR Reaction System | 20 μL: 2.0 μL 10× PCR Buffer, 2.0 μL MgCl_2_ (25mM), forward and reverse primers 1μL (10 μM) each, 2 μL dNTPs (2.5 mM), 1 μL DMSO, 2 μL DNA and 0.2 μL rTaq |
|  | PCR Reaction Process | General Procedure: 94℃, 4 min; 33× (94℃, 30 s; 52℃, 30 s; 72℃, 50 s); 72 ℃, 10 min; 16℃, end |
|  |  | Ramp Procedure: 32× (94℃, 1 min; 94℃, 30s; 62℃, 1 s; 48℃, 5 s); 65℃, 2 min; 65℃, 5 min; 16℃, end |
|  | Primers | matK3F: 5’-CGTACAGTACTTTTGTGTTTACGA-3’ (Ki-Joong Kim, Unpublished)  matK1R: 5’-ACCCAGTCCATCTGGAAATCTTGG-3’ (Ki-Joong Kim, Unpublished)  matK472F: 5’-CCCRTYCATCTGGAAATCTTGGTTC-3’ (Yu et al. 2011)  matK1248R: 5’-GCTRTRATAATGAGAAAGATTTCTGC-3’ (Yu et al. 2011)  mK390F: 5’-CGATCTATTCATTCAATATTTC-3’ (Cuénoud et al. 2002)  mtK1326R: 5’-TCTAGCACACGAAAGAAGT-3’ (Cuénoud et al. 2002)  matk5R: 5’-GTTCTAGCACAAGAAAGTCG-3’ (Ford et al. 2009)  matkxF: 5’-TAATTTACGATCAATTCATTC-3’ (Ford et al. 2009) |
| *trn*H*-*  *psb*A | PCR Reaction System | 20 μL: 2.0 μL 10× PCR Buffer, 2.0 μL MgCl_2_ (25mM), forward and reverse primers 1μL (10 μM) each, 2 μL dNTPs (2.5 mM), 2 μL DNA and 0.2 μL rTaq |
|  | PCR Reaction Process | General Procedure: 94℃, 4 min; 35× (94℃, 30 s; 53℃, 30 s; 72℃, 1 min); 72℃, 10 min; 16℃, end |
|  |  | Ramp Procedure: 94℃, 1 min; 32× (94℃, 30s; 60℃, 1s; 48℃, 5s; 65℃, 90 s); 65℃, 5 min; 16℃, end |
|  | Primers | trnH2: 5’-CGCATGGTGGATTCACAATCC-3’ (Fazekas et al. 2010) |
|  |  | trnH: 5’-CGCGCATGGTGGATTCACAATCC-3’ (Tate and Simpson 2003) |
|  |  | psbA: 5’-GTTATGCATGAACGTAATGCTC-3’ (Sang et al. 1997) |
| ITS | PCR Reaction System | 20 μL: 2.0 μL 10× PCR Buffer, 2.0 μL MgCl_2_ (25mM), forward and reverse primers 1μL (10 μM) each, 2 μL dNTPs (2.5mM), 1 μL DMSO, 2 μL BSA (1mg/ml), 2 μL DNA and 0.2 μL rTaq |
|  | PCR Reaction Process | General Procedure: 95℃, 4 min; 35× (94℃, 45 s; 56℃, 1 min; 72℃, 1 min); 72℃, 10 min; 16℃, end |
|  | Primers | ITS5P: 5’-GGAAGGAGAAGTCGTAACAAGG-3’ (Möller and Cronk 2001) |
|  |  | ITS8P: 5’-CACGCTTCTCCAGACTACA-3’ (Möller and Cronk 2001) |
|  |  | ITS4: 5’-TCCTCCGCTTATTGATATGC-3’ (White et al. 1990) |
|  |  | ITS2F: 5’-ATGCGATACTTGGTGTGAAT-3’ (Chen et al. 2010) |
|  |  | ITS3R: 5’-GACGCTTCTCCAGACTACAAT-3’ (Chen et al. 2010) |

**References**

Chen, S, Yao H, Han J, Liu C, Song J, Shi L, Zhu Y, Ma X, Gao T, Pang X, Luo K, Li Y, Li X, Jia X, Lin Y, Leon C (2010) Validation of the ITS2 region as a novel DNA barcode for identifying medicinal plant species. *PLoS ONE*, 5: e8613

Cuénoud P, Savolainen V, Chatrou LW, Powell M, Grayer RJ, Chase MW. 2002. Molecular phylogenetics of Caryophyllales based on nuclear 18S rDNA and plastid *rbc*L, *atp*B and *mat*K DNA sequences. *American Journal of Botany*, 89: 132–144.

Fay MF, Cameron KM, Prance GT, Lledo MD, Chase MW (1997) Familial relationships of *Rhabdodendron* (Rhabdodendraceae): plastid *rbc*L sequences indicate a caryophyllid placement. *Kew Bulletin*, 52: 923–932.

Fazekas AJ, Steeves R, Newmaster SG, Hollingsworth PM (2010) Stopping the stutter: improvements in sequence quality from regions with mononucleotide repeats can increase the usefulness of non-coding regions for DNA barcoding. *Taxon*, 59: 694–697.

Ford CS, Ayres KL, Toomey N, Haider N, Van Alphen Stahl J, Kelly LJ, Wikström N, Hollingsworth PM, Duff RJ, Hoot SB, Cowan RS, Chase MW, Wilkinson MJ (2009) Selection of candidate coding DNA barcoding regions for use on land plants. *Botanical Journal of the Linnean Society*, 159: 1–11.

Kress WJ, Erickson DL, Jones FA, Swenson NG, Perez R, Sanjur O, and Bermingham E (2009) Plant DNA barcodes and a community phylogeny of a tropical forest dynamic plot in Panama. *Proceedings of the National Academy of Science USA*, 106: 18621–18626.

Levin RA, Wagner WL, Hoch PC, Nepokroeff M, Pires JC, Zimmer EA, Sytsma KJ (2003) Family-level relationships of Onagraceae based on chloroplast *rbc*L and *ndh*F data. *American Journal of Botany*, 90: 107–115.

Möller M, Cronk QCB. 2001. Evolution of morphological novelty: a phylogenetic analysis of growth patterns in *Streptocarpus* (Gesneriaceae). *Evolution,* 55: 918–929.

Olmstead RG, Michaels HJ, Scott KM, Palmer JD (1992) Monophyly of the Asteridae and identification of their major lineages inferred from DNA sequences of *rbc*L. *Annals of the Missouri Botanical Garden*, 79: 249–265.

Sang T, Crawford DJ, Stuessy TF (1997) Chloroplast DNA phylogeny, reticulate evolution, and biogeography of *Paeonia* (Paeoniaceae). *American Journal of Botany*, 84: 1120–1136.

Tate JA, Simpson BB (2003) Paraphyly of *Tarasa* (Malvaceae) and diverse origins of the polyploid species. *Systematic Botany*, 28: 723–737.

White TJ, Bruns TD, Lee SB, Taylor JW (1990) Amplification and direct sequencing of fungal ribosomal RNA genes for phylogenetics. In: Innis MA, Gelfand GH, Sninsky JJ, White TJ (eds) PCR protocols: a guide to methods and applications. Academic Press, San Diego, pp 315–322.

Yu J, Xue JH, Zhou SL (2011) New universal *mat*K primers for DNA barcoding angiosperms. *Journal of Systematics and Evolution*, 49: 176-181.
